# Targeting Human Pancreatic Cancer with a Fluorophore-Conjugated Mucin 4 (MUC4) Antibody: Initial Characterization in Orthotopic Cell Line Mouse Models

**DOI:** 10.3390/jcm13206211

**Published:** 2024-10-18

**Authors:** Sunidhi Jaiswal, Kristin E. Cox, Siamak Amirfakhri, Aylin Din Parast Saleh, Keita Kobayashi, Thinzar M. Lwin, Sumbal Talib, Abhijit Aithal, Kavita Mallya, Maneesh Jain, Aaron M. Mohs, Robert M. Hoffman, Surinder K. Batra, Michael Bouvet

**Affiliations:** 1 Department of Surgery, University of California San Diego, La Jolla, CA 92093, USA; s3jaiswal@health.ucsd.edu (S.J.); siamirfakhri@health.ucsd.edu (S.A.); aylindnp@gmail.com (A.D.P.S.); kjkobayashi@ucsd.edu (K.K.); rhoffman@health.ucsd.edu (R.M.H.); 2 VA San Diego Healthcare System, La Jolla, CA 92161, USA; 3 Department of Surgical Oncology, City of Hope, Duarte, CA 91010, USA; tlwin@coh.org; 4 Department of Pharmaceutical Sciences, University of Nebraska Medical Center, Omaha, NE 68198-5870, USA; stalib@unmc.edu (S.T.); aaron.mohs@unmc.edu (A.M.M.); 5 Department of Biochemistry and Molecular Biology, University of Nebraska Medical Center, Omaha, NE 68198-5870, USA; abhijit.aithal@unmc.edu (A.A.); kmallya@unmc.edu (K.M.); mjain@unmc.edu (M.J.); sbatra@unmc.edu (S.K.B.); 6 AntiCancer Inc., San Diego, CA 92111, USA; 7 UCSD Moores UCSD Cancer Center, 3855 Health Sciences Drive #0987, La Jolla, CA 92093-0987, USA

**Keywords:** pancreatic cancer, orthotopic mouse model, fluorescent antibody, mucin 4, MUC4, tumor labeling

## Abstract

**Background/Objectives:** Pancreatic cancer is the third leading cause of death related to cancer. The only possible cure presently is complete surgical resection; however, this is limited by difficulty in clearly defining tumor margins. Enhancement of the visualization of pancreatic ductal adenocarcinoma (PDAC) tumor margins using near-infrared dye-conjugated tumor-specific antibodies was pioneered by using anti-CEA, anti-CA19.9, and anti-MUC5AC in orthotopic mouse models of pancreatic cancer. Recently, an antibody to Mucin 4 (MUC4) conjugated to a fluorescent probe has shown promise in targeting colon tumors in orthotopic mouse models. **Methods:** In the present study, we targeted pancreatic cancer using an anti-MUC4 antibody conjugated to IRDye800 (anti-MUC4-IR800) in orthotopic mouse models. Two pancreatic cancer human cell lines were used, SW1990 and CD18/HPAF. **Results:** Anti-MUC4-IR800 targeted the two pancreatic cancer cell line tumors in orthotopic mouse models with high tumor-to-pancreas ratios and high tumor-to-liver ratios, with greater targeting seen in SW1990. **Conclusions:** The present results suggest anti-MUC4-IR800’s potential to be used in fluorescence-guided surgical resection of pancreatic cancer.

## 1. Introduction

The survival rate for pancreatic ductal adenocarcinoma (PDAC) after five years is below 10% [1,2,3,4,5]. It has a grim outlook as about 80% of patients are diagnosed with either locally advanced or metastatic disease [6,7,8]. The only possibility for a cure is complete surgical resection. Evaluating margins is crucial as microscopic tumor-positive margins (R1 resection) are found in as many as 70% of cases, which may lead to tumor recurrence [9,10,11,12]. It is also important to identify small-volume peritoneal implants or liver metastases that would preclude a major pancreatic resection. Therefore, there is an important requirement for new intraoperative imaging techniques to improve the visualization of tumor margins and metastases [13,14]. 

Near-infrared (NIR) fluorescence imaging can differentiate tumor tissue from normal surrounding tissues, thus enhancing survival after surgery [15,16,17,18]. In recent years, fluorescence-guided surgery (FGS) utilizing near-infrared imaging has emerged as a pioneering and promising optical imaging method to better define the tumor margins and thus improve survival after surgery [19,20,21,22,23,24]. FGS requires an imaging system capable of exciting a fluorophore linked to a tumor-targeting molecule administered intravenously and capturing its emission [25,26,27,28]. Different tumor-specific antibodies conjugated to fluorophores, including anti-CEA [29], anti-CA19.9 [30], and anti-MUC5AC [31], have been used to target pancreatic cancer in orthotopic mouse models. These fluorescently-labeled antibodies demonstrated accurate localization of tumor margins.

Mucins are recognized as multifunctional glycoproteins, and coat epithelial cell surfaces within the gastrointestinal tract, which is important for the lubrication and protection of the gut from pathogens [32,33]. Additionally, mucins also take part in the pathogenesis of pancreatic cancer and act as both targets and diagnostic biomarkers for therapy [34]. Mucins are identified by the first three letters “MUC” followed by a number indicating the encoding gene. Mucins are divided in two categories: secreted and membrane-bound [34].

Mucin 4 (MUC4) belongs to the membrane-bound mucins category and has high expression in pancreatic cancer. Its participation in driving the disease’s pathobiology and aggressiveness has been well recognized [35,36]. While normal pancreatic ducts show no detectable expression at the initial stage of pancreatic intraepithelial neoplasia, MUC4 becomes identifiable and its expression steadily rises as the disease progresses towards PDAC [37]. MUC4 expression triggers neoplastic transformation [38], enhances both tumorigenesis and metastasis, and influences the interaction and communication between components of the tumor microenvironment and cancer cells [38,39]. 

MUC4 antibody conjugated with a fluorescent dye was thought to be a good candidate for labeling pancreatic cancer. The present study describes the bright and selective labeling of pancreatic cancer in orthotopic mouse models implanted with the human pancreatic cancer cell lines SW1990 and CD18/HPAF using fluorescent MUC4 antibodies.

## 2. Materials and Methods

### 2.1. Mice

4-to-6-week-old male and female athymic nude mice were purchased from The Jackson Laboratory (Bar Harbor, ME, USA). Equal numbers of male and female mice were used in this study. All mice were kept in an animal facility and provided with autoclaved water and food. Before any surgical procedure, mice were anesthetized with a 150–250 µL cocktail containing ketamine, xylazine, and phosphate-buffered saline (PBS), via intraperitoneal (IP) injection. Mice also received buprenorphine diluted in PBS (dosage: 0.05 mg/kg) via subcutaneous injection for postoperative pain control after surgery. Mice were sacrificed by CO_2_ inhalation followed by cervical dislocation at the end of study.

### 2.2. Cell Culture

The human pancreatic cancer cell lines SW1990 and CD18/HPAF were obtained from Dr. Surinder K. Batra’s laboratory and maintained in Dulbecco’s Modified Eagle Medium (DMEM) with high-glucose as previously reported [40]. The medium was supplemented with 10% FBS and 1% penicillin–streptomycin. The cells were maintained at 37 °C and CO_2_ at 5%. Regular cell passaging was performed to maintain optimum cell growth.

### 2.3. Establishment of Pancreatic Cancer Cell Line Xenografts

Nude mice were first injected with a ketamine cocktail to be anesthetized. When mice were completely anesthetized, 1 × 10^6^ SW1990 or CD18/HPAF cells were injected subcutaneously in the bilateral flanks. After 2–3 weeks, subcutaneous tumors reached approximately 1 cm in size, and were used for further passaging. For passaging, tumors were collected and divided into 1 mm^3^ fragments and implanted into additional mice, subcutaneously, in four flanks. 

### 2.4. Orthotopic Pancreatic Cancer Xenograft Models

Surgical orthotopic implantation (SOI) was used to establish an orthotopic pancreatic cancer mouse model as previously described [29]. Briefly, first, a 70% ethanol solution was used to disinfect the ventral surface of anesthetized mice. To enter the abdominal cavity, a small incision was made on the left flank of the anesthetized mouse. Then, 8-0 nylon sutures (Ethicon Inc., Sommerville, NJ, USA) were used to attach a single 1 mm^3^ SW1990 or CD18/HPAF tumor fragment to the middle of the pancreas. The pancreas implanted with the tumor fragment was placed back into the abdomen, and the incision was closed using 6–0 vicryl sutures. Postoperative pain was managed with the subcutaneous administration of 100 µL buprenorphine solution as described above. 

### 2.5. Antibody Conjugation

Antibodies to MUC4 (anti-MUC4) and the mouse IgG isotype control were conjugated to the IRDye800 dye based on a method previously reported [41]. Briefly, the IRDye800 CW protein labeling kit (LICOR Biosciences, Lincoln, NE, USA) was used for conjugation. 100 µL of potassium phosphate buffer, pH 9, was added to 1 mg of anti-MUC4 (1 mg/mL) or IgG to raise the pH to 8.5. The dye (0.1 mg) was dissolved in 50 µL of nanopure water. Then, 12 µL of the dye were added to the antibody solution and incubated for 2 h in the dark at room temperature. Free dye was removed from the conjugate using a Zeba desalting column (Thermo Fisher Scientific, Waltham, MA, USA). The dye-to-protein (D/P) ratio and protein concentration were quantified using spectra obtained with an Evolution 220 absorbance spectrophotometer (Thermo Fisher Scientific, Waltham, MA) [41]. The fluorescence spectrum of the conjugate was obtained using a FluoroMax 4 spectrofluorometer (Horiba Scientific; Irvine, CA, USA). Sodium dodecyl sulfate–polyacrylamide gel electrophoresis (SDS-PAGE) was performed to confirm the conjugation. IRDye800 conjugated to anti-MUC4 (anti-MUC4-IR800) or IRDye800 conjugated to IgG (IgG-IR800), IRDye800 was loaded on a 4–20% gradient gel (Bio-Rad, 4568094, Hercules, CA, USA) and electrophoresis was performed at 90–125 V. Then, the gels were imaged using an 800 nm channel on a LI-COR Odyssey M imaging system (LI-COR Biosciences, Lincoln, NE).

### 2.6. Antibody Conjugate Administration

50 µg anti-MUC4-IR800 (n = 5) or 50 µg IgG-IR800 control (n = 4) were administered via tail vein injection into orthotopic nude mouse models of SW1990 and CD18/HPAF. After 72 h, the mice were sacrificed, and a midline laparotomy was performed for bright-light and NIR imaging.

### 2.7. Near-Infrafred Imaging and Fluorescence Image Analysis

A Pearl Trilogy Small Animal Imaging System (LI-COR Biosciences, Lincoln, NE, USA), with 785 nm excitation and 820 nm emission (800 channel), was used for near-infrared (NIR) imaging. Bright-light images were obtained using the white light channel. Areas of interest were drawn around the tumors, normal pancreas, and liver with the system’s software, while looking at the images using the bright-light channel. The imaging system then evaluated the mean fluorescence intensity (mFI) of the NIR signal of areas of interest drawn around the tumor, normal pancreas, and liver. Tumor-to-normal pancreas (TPR) and tumor-to-liver (TLR) ratios were calculated by dividing the mFI of the tumor by the mFI of the normal pancreas for TPR and by the mFI of the liver for TLR. 

### 2.8. Biodistribution

Following imaging of the tumors as above, necropsy was performed, and the tumor, normal pancreas, liver, spleen, stomach, cecum, kidney, lung, and ear were collected and imaged using the 800 channel of Pearl Trilogy Small Animal Imaging System to obtain NIR fluorescence biodistribution data. Areas of interest were drawn around collected tissues and the tumors by viewing the bright-light images. The mFI within the area of interest was calculated using the 800 nm channel of the Pearl System.

### 2.9. Immunohistochemistry

Orthotopic tumors were collected during mouse necropsy. Collected orthotopic tumors were preserved in formalin for a minimum of 72 h before placing them in paraffin cubes, which were then sectioned. Hematoxylin and eosin (H&E) staining was performed on sectioned slides following standard protocols. Immunohistochemistry was carried out according to established procedures. Briefly, serial sections were incubated with anti-MUC4 antibody (2 µg/mL) or IgG isotype control antibody (2 µg/mL) overnight at 4 °C followed by incubating with a peroxidase-labeled secondary antibody, and subsequent detection of the signal using the DAB substrate (Vector Universal Staining kit) (VectorLab, Newark, CA, USA) was then carried out. 

## 3. Results

### 3.1. Antibody Conjugate Characterization

**Figure 1:**
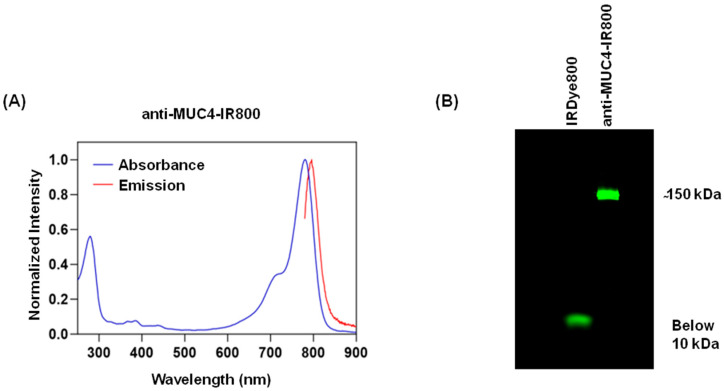
Characterization of anti-MUC4-IR800 conjugates. (**A**) Excitation and emission spectra of the anti-MUC4-IR800 conjugate. (**B**) SDS gel showing a fluorescent band below 10 kDa for the free IRDye800 and a ~150 kDa band for anti-MUC4-IR800. The dye/protein ratio is 1.42.

Anti-MUC4 and the mouse IgG isotype control were conjugated to the IRDye800 dye by forming a stable amide bond between the free amines of anti-MUC4 or IgG and the NHS ester group of the dye. The conjugates of the anti-MUC4 antibody and the mouse IgG isotype control with IRDye800 were characterized by their excitation/emission profile and the SDS gels shown in Figure 1 and Figure S1. When IRDye800 and anti-MUC4-IR800 or IgG-IR800 were electrophoresed on SDS gel under non-reducing conditions, IRDye800 alone demonstrated a fluorescent band near the dye front, and anti-MUC4-IR800 showed fluorescence by the 150 kD molecular weight marker, demonstrating the purity of the conjugated antibody (Figure 1B and Figure S1A). Similar results were observed for IgG-IR800 (Figure S1B). The dye-to-protein ratios observed for anti-MUC4-IR800 and IgG-IR800 were 1.42 and 1.04, respectively. 

### 3.2. Targeting Pancreatic Orthotopic Cell Line Tumors with Anti-MUC4-IR800

**Figure 2:**
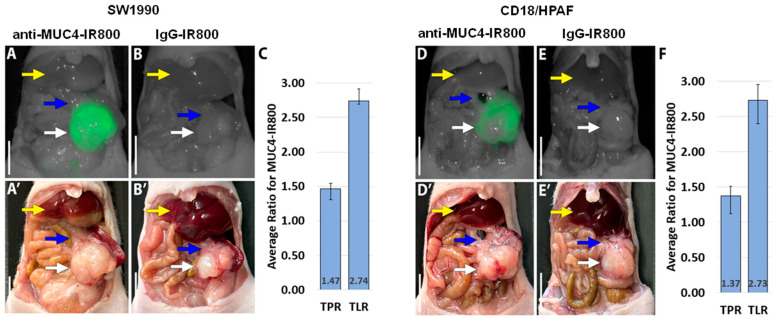
Orthotopic models of the SW1990 (left panel) and CD18/HPAF human pancreatic cancer cell lines (right panel). (**A**) NIR and (**A’**) bright-light images of SW1990 orthotopic nude mouse models labeled with 50 µg anti-MUC4-IR800. (**B**) Non-specific NIR labeling with 50 µg IgG-IR800 and (**B’**) bright-light images. (**C**) Average TPR and TLR of mice treated with 50 µg anti-MUC4-IR800, n = 5. (**D**) NIR and (**D’**) bright-light images of CD18/HPAF orthotopic nude mouse models labeled with 50 µg anti-MUC4-IR800. (**E**) Non-specific NIR labeling with 50 µg IgG-IR800 and (**E’**) bright light images. (**F**) Average TPR and TLR of mice treated with 50 µg anti-MUC4-IR800, n = 5. Scale bar: 1 cm. White arrow: pancreatic tumor; blue arrow: normal pancreas; yellow arrow: liver. Error bars represent the standard error.

Anti-MUC4-IR800 brightly distinguished SW1990 and CD18/HPAF tumors from the normal pancreas (Figure 2A,D). In contrast, in their respective models, SW1990- and CD18/HPAF-bearing mice treated with IgG-IR800 showed minimal non-specific fluorescence intensity in the tumors (Figure 2B,E). The average mean fluorescence intensity (mFI) for SW1990 tumors was 0.848 (SE ± 0.065) for mice treated with anti-MUC4-IR800 (Table 1) and 0.194 (SE ± 0.013) for mice injected with IgG-IR800 (Table S1). The average mFI for CD18/HPAF tumors was 1.049 (SE ± 0.066) for mice injected with anti-MUC4-IR800 (Table 2) and 0.273 (SE ± 0.024) for mice injected with IgG-IR800 (Table S2). Tumor-to-pancreas and tumor-to-liver ratios (TPR and TLR) were then calculated for each cell line (SW1990 and CD18/HPAF) and each probe (anti-MUC4-IR800 and IgG-IR800). In SW1990, anti-MUC4-IR800 had an average TPR of 1.47 (SE ± 0.078) and an average TLR of 2.74 (SE ± 0.174). In CD18/HPAF, anti-MUC4-IR800 had an average TPR of 1.37 (SE ± 0.130) and an average TLR of 2.73 (SE ± 0.220) (Table 2 and Table S2).

### 3.3. Flourescence Biodistribution

**Figure 3:**
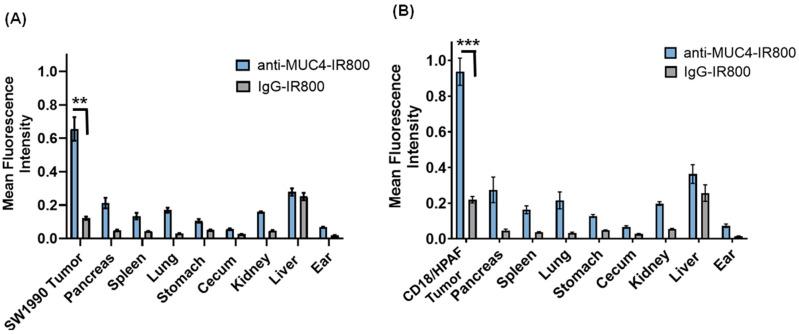
Fluorescence biodistribution of anti-MUC4-IR800 in different organs in (**A**) SW1990, n = 5 (anti-MUC4-IR800), n = 4 (IgG-IR800), and (**B**) CD18/HPAF tumor-bearing mice, n = 5 (anti-MUC4-IR800), n = 5 (IgG-IR800), at 72 h post-administration. Error bars represent the standard error. ** *p*-value = 0.0008. *** *p*-value = 0.0077.

Following imaging of the pancreatic tumors, necropsy was performed to allow the collection of fluorescence biodistribution data. In mice that received anti-MUC4-IR800, the highest mFI values were seen in the tumors of both SW1990 (0.655 ± 0.158)- and CD18/HPAF (0.936 ± 0.076)-bearing mice (Figure 3). In the mice that received IgG-IR800, the liver demonstrated the highest mFI with values of 0.251 (SE ± 0.022) and 0.256 (SE ± 0.046) for the SW1990 and CD18/HPAF orthotopic models, respectively (Figure 3).

### 3.4. Confirmation of MUC4 Expression in Tumors by Immunohistochemistry

**Figure 4:**
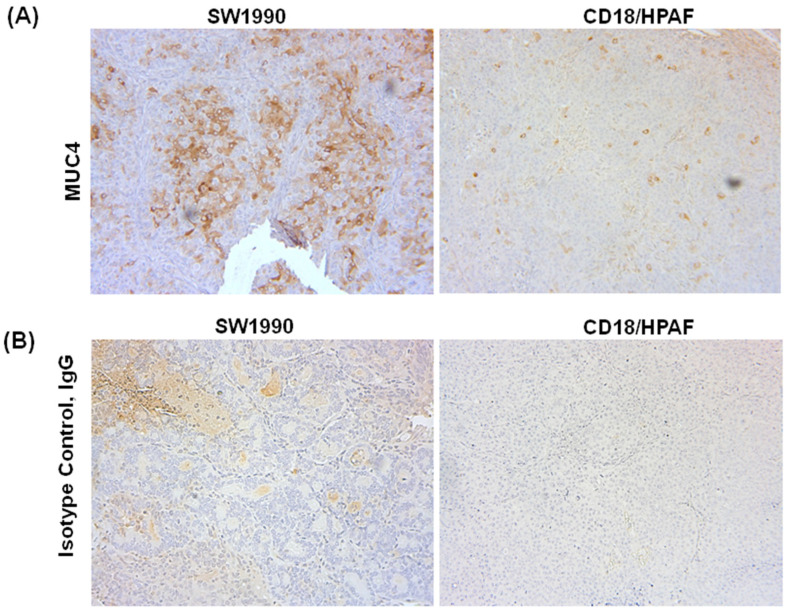
Immunohistochemical staining of MUC4 and IgG isotype control in orthotopic xenografts of pancreatic cancer cell lines: (**A**) 10× images of SW1990 and CD18/HPAF tumor sections showing MUC4 expression (brown staining); (**B**) 10× images of SW1990 and CD18/HPAF tumor sections treated with the isotype control, IgG antibody.

Anti-MUC4 antibody staining was more prominent in the SW1990 orthotopic tumors (Figure 4A) compared to CD18/HPAF tumors (Figure 4B). However, the anti-MUC4-IR800 probe was still able to target the tumor types equally compared to surrounding tissues, suggesting that high expressions of MUC4 may not be necessary to effectively use the anti-MUC4 antibody. Tumors treated with the isotype control IgG antibody showed no specific staining (Figure 4B).

## 4. Discussion

Currently, the only curative treatment for pancreatic cancer is surgical resection, which makes achieving R0 resection crucial [42,43]. The surgeon mainly relies on visualization and tactile cues to determine the appropriate resection margins and ensure complete tumor resection. Surgical navigation in real time with fluorescence guidance has improved the ability of surgeons to visualize the tumor and may help to achieve more oncologically complete resections. 

There is increasing interest in the use of mucins to target PDAC, with a number of comprehensive reviews recently published on this topic [44,45]. Pancreatic carcinogenesis is marked by changes in mucin expression patterns at various stages of tumor progression. While MUC4 is not expressed in the normal pancreas, as confirmed in the present study, 83-89% of PDAC tumors express MUC4 [37,45]. Increasing MUC4 expression has been observed through the various stages of carcinogenesis, with MUC4 expression occuring as early as the PanIN1A stage [45]. The lack of MUC4 expression in the normal pancreas and its expression in early stages of pancreatic cancer make it an excellent tumor-specific target for use in FGS as well as a potential tumor biomarker.

Previously, we showed that MUC5AC-IR800 could brightly and specifically label the primary tumor and abdominal wall metastases with an average tumor-to-pancreas ratio of 2.183 (±0.619) in a patient-derived orthotopic xenograft model of pancreatic cancer (AA1305) [31]. In the present study, we demonstrated that fluorescent anti-MUC4-IR800 can selectively target pancreatic cancers in orthotopic cell line mouse models compared to both normal pancreatic tissue and the liver. Evaluating the probe specificity for tumors compared to the liver is important as the liver is a common site for pancreatic cancer metastases. High tumor-to-liver ratios of 2.74 (±0.174) and 2.73 (±0.223) in the SW1990 and CD18/HPAF orthotopic models, respectively, were obtained in the present study. Clinical trials of FGS in pancreatic cancer have successfully identified cancerous lesions with ratios as low as 1.3 [46]. 

Immunohistochemical staining of SW1990 and CD18/HPAF tumors with an anti-MUC4 antibody confirmed high expression of MUC4 in SW1990 and lower expression in CD18/HPAF tumors. However, anti-MUC4-IR800 could still image and localize the CD18/HPAF tumors despite the lower expression levels of MUC4. MUC4 may be more accessible to anti-MUC4IR800 in live CD18/HPAF tumors than SW1990 tumors, accounting for the greater fluorescence of CD18/HPAF. This will be further investigated in future studies.

The present study used anti-MUC4-IR800 in two human pancreatic cancer cell line orthotopic mouse models to demonstrate specific labeling of human pancreatic cancer. Figure 2 of the manuscript shows clear florescence intensity differences between the tumor and the normal pancreas with a tumor-to-normal pancreas ratio of 1.37–1.47 after MUC4-IR800 targeting. These ratios suggest that fluorescence due to MUC4-IR800 targeting could allow the surgeon to better visualize margins during a pancreatectomy and reduce the R1 resection rate. In addition, anti-MUC4-IR800 targeting can facilitate visualizing margins for residual disease much more rapidly than sending the specimen to the pathologist intraoperatively. Surgical margins in pancreatic cancer surgery are of particular importance as these include the bile duct, pancreatic neck, and retroperitoneal margin with the portal vein.

The present study suggests that anti-MUC4-IR800 has the potential to improve the detection of pancreatic cancer in the clinic. Cetuximab and panitumumab conjugated to IRDye800 have been used in clinical trials [47,48,49,50,51,52]. Neither had any serious side effects, suggesting IRDye800 is safe to use in patients. Future studies will use patient-derived orthotopic xenograft (PDOX) models of pancreatic cancer to validate tumor targeting using anti-MUC4-IR800 for subsequent translation into the clinic.

## 5. Conclusions

This present study demonstrated that anti-MUC4 antibodies conjugated to near-infrared dyes offer precise in vivo labeling of pancreatic cancer in orthotopic mouse models. This tumor-specific MUC4-IR800 fluorescent antibody shows promise as a potential clinical tool to enhance resection margins and overall survival rates in pancreatic cancer patients.

## Figures and Tables

**Table 1:** Mean fluorescence intensity values of the orthotopic tumors, normal pancreas, and liver for individual mice bearing a SW1990 tumor injected with anti-MUC4-IR800. Calculated tumor-to-liver ratios and tumor-to-pancreas ratios. SE: standard error.

Mouse	Tumor (mFI)	Normal Pancreas (mFI)	Liver (mFI)	Tumor/Liver (TLR)	Tumor/Pancreas (TPR)
1	0.830	0.569	0.304	2.73	1.458
2	0.709	0.450	0.292	2.43	1.575
3	1.000	0.599	0.333	3.00	1.669
4	0.708	0.496	0.308	2.30	1.427
5	0.997	0.829	0.308	3.24	1.202
Average (±SE)	0.848 (±0.065)	0.589 (±0.065)	0.309 (±0.006)	2.74 (±0.174)	1.47 (±0.078)

**Table 2:** Mean fluorescence intensity values of the orthotopic tumors, normal pancreas, and liver for individual mice bearing a CD18/HPAF tumor injected with anti-MUC4-IR800. Calculated tumor-to-liver ratios and tumor-to-pancreas ratios. SE: standard error.

Mouse	Tumor (mFI)	Normal Pancreas (mFI)	Liver (mFI)	Tumor/Liver (TLR)	Tumor/Pancreas (TPR)
1	0.963	0.816	0.426	2.26	1.18
2	0.880	0.490	0.334	2.63	1.80
3	1.060	0.677	0.3240	3.27	1.57
4	1.320	1.060	0.408	3.24	1.25
5	1.020	0.946	0.451	2.26	1.08
Average (±SE)	1.049 (±0.066)	0.798 (±0.100)	0.389 (±0.025)	2.73 (±0.220)	1.37 (±0.130)

## Data Availability

The original contributions presented in the study are included in the article/Supplementary Materials; further inquiries can be directed to the corresponding author/s.

## Supplementary Materials

The following supporting information can be downloaded at https://www.mdpi.com/article/10.3390/jcm13206211/s1: Figure S1: Characterization of anti-MUC4-IR800 and IgG-IR800 conjugates. (A) SDS gel showing the fluorescent band below 10 kDa for IRDye800 and ~150 kDa for the anti-MUC4-IR800 conjugate. The dye/protein ratio is 1.42. (B) SDS gel showing a fluorescent band below 10 kDa for IRDye800 and one at ~150 kDa for the IgG-IR800 conjugate. The dye/protein ratio is 1.04. Table S1: Mean fluorescence intensity of orthotopic tumors, pancreas and liver for individual mice bearing SW1990 tumors, treated with non-specific IgG-IR800 antibody. Calculated tumor-to-liver ratios (TLRs) and tumor-to-pancreas ratios (TPRs). SE: standard error. Table S2: Mean fluorescence intensity of orthotopic tumor, pancreas, and liver for individual mice bearing CD18/HPAF tumors, treated with the non-specific IgG-IR800 antibody. Calculated tumor-to-liver ratios (TLRs) and tumor-to-pancreas ratios (TPRs). SE: standard error.
