# Supplementary material for: Targeting Human Pancreatic Cancer with a Fluorophore-Conjugated Mucin 4 (MUC4) Antibody: Initial Characterization in Orthotopic Cell Line Mouse Models
Source: J Clin Med. 2024 Oct 18;13(20):6211. doi: 10.3390/jcm13206211 (PMC11508345; doi:10.3390/jcm13206211)
Supplement: Supplementary file 1 [file jcm-13-06211-s001.zip › jcm-3207446-supplementary.pdf]

## Supplemental Materials

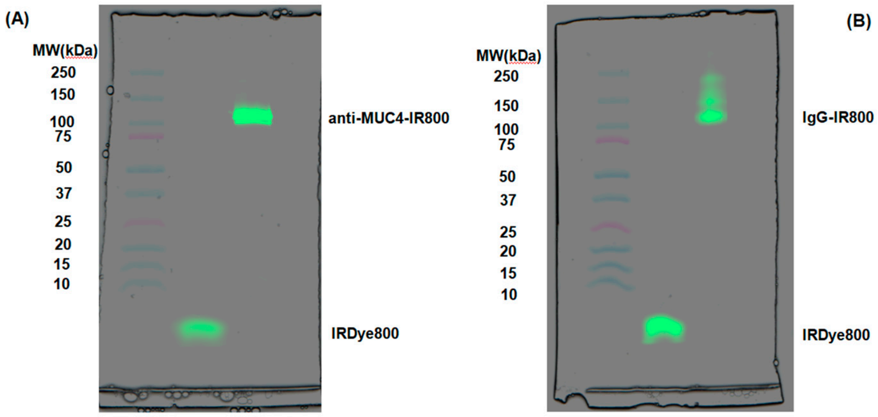


**Supplementary Figure S1.** Characterization of anti-MUC4-IR800 and IgG-IR800 conjugations. (A) SDS gel showing the fluorescent band below 10 kDa for IRDye800 and ~150 kDa for the anti-MUC4-IR800 conjugate. Dye/Protein ratio 1.42. (B) SDS gel showing the fluorescent band below 10 kDa for IRDye800 and ~150 kDa for the IgG-IR800 conjugate. Dye/Protein ratio 1.04.

**Supplementary Table S1:** Mean fluorescence intensity (mFI) values of orthotopic tumors, normal pancreas, and the liver for individual mice bearing SW1990 tumors, treated with IgG-IR800. Calculated tumor-to-liver ratios (TLR) and tumor-to-pancreas ratios (TPR). SE: Standard Error.

| Mouse | Tumor (mFI) | Normal Pancreas (mFI) | Liver (mFI) | Tumor/Liver (TLR) | Tumor/Pancreas (TPR) |
|---|---|---|---|---|---|
| **1** | 0.194 | 0.103 | 0.188 | 1.03 | 1.88 |
| **2** | 0.181 | 0.135 | 0.200 | 0.91 | 1.34 |
| **3** | 0.233 | 0.160 | 0.278 | 0.84 | 1.46 |
| **4** | 0.169 | 0.085 | 0.165 | 1.02 | 1.99 |
| **Average(±SE)** | 0.194(±0.013) | 0.121(±0.016) | 0.208(±0.040) | 0.95(±0.047) | 1.67(±0.157) |

**Supplementary Table S2:** Mean fluorescence intensity (mFI) values of orthotopic tumors, normal pancreas, and the liver for individual mice bearing CD18/HPAF tumors, treated with IgG-IR800. Calculated tumor-to-liver ratios (TLR) and tumor-to-pancreas ratios (TPR). SE: Standard Error.

| Mouse | Tumor (mFI) | Normal Pancreas (mFI) | Liver (mFI) | Tumor/Liver (TLR) | Tumor/Pancreas (TPR) |
|---|---|---|---|---|---|
| 1 | 0.265 | 0.185 | 0.240 | 1.10 | 1.43 |
| **2** | 0.341 | 0.161 | 0.269 | 1.27 | 2.12 |
| **3** | 0.305 | 0.123 | 0.201 | 1.52 | 2.48 |
| **4** | 0.262 | 0.230 | 0.294 | 0.89 | 1.14 |
| **5** | 0.192 | 0.142 | 0.069 | 2.78 | 1.35 |
| **Average(±SE)** | 0.273(±0.024) | 0.168(±0.018) | 0.215(±0.039) | 1.51(±0.332) | 1.70(±0.253) |
